# Femi Oyebode

**DOI:** 10.1192/bjb.2020.123

**Published:** 2021-02

**Authors:** Abdi Sanati

**Figure:**
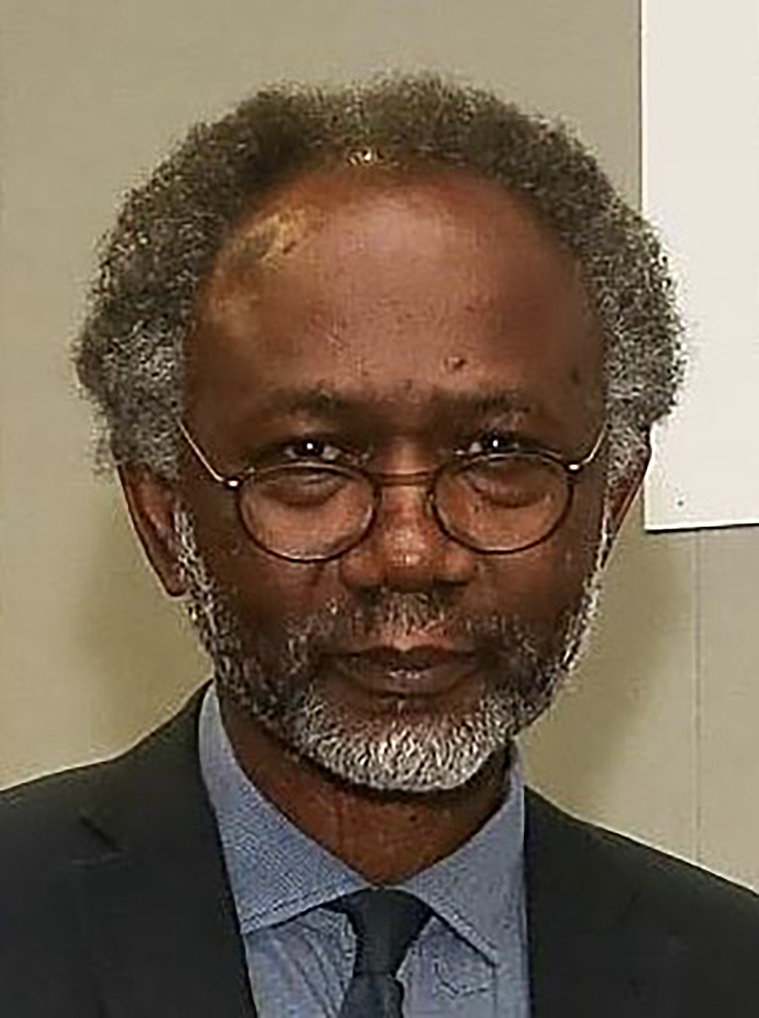

Professor Femi Oyebode does not need any introduction. He is one of our greatest experts in the field of descriptive psychopathology. In recent years, he has taken over the influential psychopathology textbook *Sims’ Symptoms in the Mind*. He is also an authority on delusional misidentification syndromes and rare psychiatric disorders. As someone who is interested in psychopathology, I have always found Professor Oyebode's writing very informative and an amazing read. I finally met him a few years ago at the International Congress of the Royal College of Psychiatrists. He has also edited a book on literature and psychiatry (*Mindreadings: Literature and Psychiatry*), written one on the theatre (*Madness at the Theatre*) and published six volumes of poetry.

**Professor Oyebode, first I wanted to thank you for your time for this interview during the lockdown! Having read your work, I wanted to start the interview with a question on the humanities. How important do you think the humanities are for psychiatry?**

I think the humanities are important for the whole of medicine, and not just psychiatry. The medical humanities include moral philosophy, ethics, medical history and literature, among others. I focus on literature, which is my expertise. The aim of literature in medical humanities is to help us grasp the living experience of others. It can help us with the living experience of our patients too. We can emphasise the subjective experience of our patients using literature as an entry point.

**Do you think reading fiction can help psychiatrists to improve their understanding of their patients’ narratives, especially in the era of assembly-line psychiatry, when the narratives are lost in the rigid system?**

It is a complex question and I will try to answer it in a roundabout way. If one is thinking of the best use of literature for psychiatry, I would recommend reading the autobiographies or memoirs of people who have suffered from mental illness. Even more so when the author is a professional writer. Writers have the gift of language to describe complexities of those experiences in words. Being familiar with those descriptions would help the psychiatrist to communicate better in their practice. Going back to fiction, I think you were right to bring it up. It is an interesting human activity. One of fiction's tasks, if I might put it like that, is to help us live the lives we have not experienced ourselves. It engages our brains and inner life to understand the reality we have not experienced and the possibilities that come out of that reality. Fiction enables us to appreciate a multiplicity of contexts without the need to experience them directly.

**I think fiction is also important in helping us understand moral dilemmas. I have to admit I found more moral lessons in Victor Hugo's *Les Miserables* than in textbooks!**

I agree that fictional accounts are important. My example is Albert Camus’ *The Plague*. It is a multilayered and complex work. At one level, it is about Oran, a city in northern Algeria, facing the challenge of a plague epidemic. At another level, it is a metaphor for the Resistance in Nazi-occupied France during the Second World War. It explores the nature of freedom and the difficulties of choices one has to make. In the struggle with the COVID-19 pandemic we have faced similar choices – choices that include risking our lives as healthcare professionals while going out to work, which we do every day. Fiction has the power to help us see these choices and understand their complexities.

**What do you think about the role of visual arts in helping us understand the human condition?**

I have to admit I am not an expert in painting but as a West African I know a bit about sculpture. Nevertheless, I agree that the visual arts can be helpful. For example, when we look at a painting that captures the instant and fixes it in time, we can see details that we may miss on a casual, cursory view. Again, looking at a painting can potentially help us to understand human gestures and bodily attitudes and to see these in more detail. It sharpens the eye and enables us, in the clinic, to recognise postures and see subtleties we would not usually see.

**As you know, I am the chair of the Philosophy Special Interest Group at the Royal College of Psychiatrists. One reason psychiatry attracted me was the philosophical issues embedded in it. Do you think we should teach philosophy in training?**

It is not an easy subject to teach! It is not well known, but I have a PhD in Philosophy of Mind, so I am familiar with the academic side of philosophy and the teaching of philosophy. As psychiatry is conceptual, we need to think clearly and listen intensely. And philosophy could help. However, I am not sure how easy it is to teach. In addition, many philosophers of psychiatry seem not to understand that psychiatry is a practice and is much more than mere concepts and ideas. You have to deal with the facts before you can make decisions. You cannot only take time to think. Philosophy benefits psychiatry immensely with regard to conceptual reasoning but I don't recommend formal teaching in the same way that I don't recommend formal teaching of literature in psychiatry training and having exams for it. Both subjects are very enriching but when we try to teach them they can feel very dry. For that reason, I doubt that we need to have formal teaching on them.

**I do agree that we need to increase the interest in philosophy and literature and hopefully we will succeed. I am interested in your comment on philosophers without clinical experience. Looking at the philosophy of psychiatry's literature on delusions, you encounter several discussions on Capgras delusion, which is rare. I always wondered what is the reason for this preoccupation with Capgras delusion. There is also another issue when non-medics try to explain to me how medicine works. My colleague Dr Jonathan Hurlow coined the term ‘non-medicsplaining’ for this!**

Yes, it is because when you come from purely thinking professions and you don't practise psychiatry, you might not know that in psychiatry, thinking is reactive to and constrained by the facts before you. If you don't do it properly, and in time, the patient would come to harm.

**What do you think of the status of psychopathology in training? One of the reasons that I became interested in psychiatry was reading Andrew Sims’ *Symptoms in The Mind,* which you are in charge of now. It saddens me to see psychopathology is somehow side-lined in training.**

I think psychopathology is the heart of psychiatry and it is the case wherever you go. You need to know the phenomena you encounter in the clinical setting. Some make a mistake in comparing it to anatomy, as something that is already done and completed. It is an error to think that psychopathology is just a collection of definitions. It is a living and dynamic subject. This can pose a problem in writing about it. People always look for certainty, whereas psychopathology is dynamic and there is always some uncertainty in it. What I do is start with some definitions and then elaborate to show how complex it is.

**In the world of what I call assembly-line psychiatry, patients move from team to team and from practitioner to practitioner and there is hardly any chance to make a proper psychopathological examination. What is your opinion on that?**

I agree with you that the American DSM approach is very destructive, as it treats psychiatry like baking a cake and involves certain boxes to be ticked and certain recipes to be followed. It is a very basic approach. For example, in depression, the person must suffer from low mood for a certain period of time. But what is important is what is the nature of the experience of low mood. What does it consist in? What is it the person is feeling that we refer to as low mood? And, is it merely a variant of sadness or something intrinsically distinct? The checklist approach has weakened psychiatry.

**When we talk of psychopathology we mainly focus on the Western tradition. What about psychopathology from non-Western countries? I remember in 2008 when I was in Accra, I attended the African Association of Psychiatrists and Allied Professionals. I have to say that they were very inclusive and had a patient as a keynote speaker. In that conference a colleague from Uganda, Dr Catherine Abbo, had research in which she translated case vignettes from textbooks and presented to local people in villages. Interestingly, they had local names for schizophrenia and mania. For depression, they said the person was ‘thinking too much’.**

I did some work in this area many years ago. I have to add I didn't want to be pigeonholed as a cultural psychiatrist! Nevertheless, I wrote that there are deep problems with translation of words and technical terms. The translation of emotion terms can be problematic even across Western languages, which have a linguistic affinity. In English psychiatry, the term ‘anxiety’ is thought of as a psychological term. However, the word ‘anxiety’ derives from ‘angst’, which is originally a German word. In German, *angst* refers the feeling of choking. Surprisingly, a term that we take to mean an emotional experience actually refers to a physical experience. This means that we always need to be attentive to the concept that is sitting inside the word. Take the Yoruba language. If you speak Yoruba and want to translate it into English, the word for sadness, *Ìrònú*, can be translated literally as thinking hard or inner pain. But a Yoruba person does not think a person who is sad is thinking hard or has inner physical pain – he spontaneously understands that the term refers to sadness. In English, we think of mood as measured on a vertical axis, either up or down. But this notion makes no sense in Yoruba. The important question is whether the actual subjective experiences, across cultures, are similar or not, given the diversity of concepts underlying the language terms. Edward Sapir and Benjamin Whorf believed that language structured experience. This thesis of theirs is controversial and no longer accepted. For example, Eskimos have several words for snow. But does that mean that they can distinguish between different types of snow better than other people? I think when you have words for something it is probably easier to discriminate. The Western dominant view has probably influenced the way we experience the world.

**That shows there is a good case for inviting more people from the non-Western world to our conferences.**

The problem is money! In an ideal world that would be brilliant. We could have proper communication based on willingness to discuss.

**Hopefully, with the development of technology we can do it more online.**

It could be, but there are still problems. I am finishing the new edition of *Symptoms in the Mind*. My current content editor lives in India and we were scheduled to have a meeting online but the electricity there was cut off. There is still a disparity of access to resources that prevents optimal online communication.

**I am glad that you are still writing new editions of *Symptoms in the Mind*. It is an important book for professionals and trainees alike.**

I want to do one more. Currently, I am writing the seventh edition. Everything in life depends on health and I am not as young as I was. I am also writing a book on rare psychiatric symptoms.

**Perhaps you need an understudy to learn and take over. That person has big shoes to fill! Thank you very much for your time.**

